# Lifelong solid food refusal in an 8-year-old girl with a choking phobia: a case report

**DOI:** 10.1186/s40337-026-01673-z

**Published:** 2026-06-04

**Authors:** Daphna M. Finn, Chelsie N. Giambrone, Cosmina S. Ciobanu, Lisa D. Adler, Shavon C. Moore, Mary Ellen Trunko, Ana L. Ramirez

**Affiliations:** 1 UC San Diego Health Eating Disorders Center for Treatment and Research, University of California, 4510 Executive Drive Suite 315, San Diego, CA 92121 USA; 2 The Center for CBT and Mindfulness, Vista, CA USA

**Keywords:** Avoidant/restrictive food intake disorder, Choking phobia, Fear of aversive consequences, Psychopharmacology, Cognitive behavioral therapy for ARFID

## Abstract

**Background:**

This case report describes the presentation and clinical care of an 8-year-old girl treated in an eating disorder partial hospitalization program (PHP) for Avoidant/Restrictive Food Intake Disorder (ARFID), fear of aversive consequences subtype, who had never transitioned from liquids and purees to solid food.

**Case presentation:**

The patient’s choking fears manifested as complete avoidance of solid foods, maladaptive mealtime behaviors, marked food selectivity, and significant emotional, cognitive, and interoceptive distress. This report describes the successful use of multimodal therapy, including Family-Based Treatment (FBT) and Cognitive Behavioral Therapy for ARFID (CBT-AR), adapted for a higher level of care and delivered using an inhibitory learning-informed exposure framework, in combination with psychopharmacologic treatment.

**Conclusions:**

This case highlights the applicability of the inhibitory learning approach to exposure therapy within CBT-AR for pediatric choking phobia and underscores the value of PHP-level of care for younger children with severe ARFID. It also suggests that targeting anxiety, appetite, and cognitive rigidity with psychopharmacological treatments may increase food approach and interest in patients with specific food- and eating-related phobias. Finally, the report illustrates the successful treatment of an atypically severe, longstanding illness and offers clinical guidance for similar cases.

## Background

Avoidant/Restrictive Food Intake Disorder (ARFID) presents a multidimensional model of food refusal, which includes food avoidance due to sensory sensitivity or “picky eating,” fear of negative consequences, and low interest or lack of hunger [1]. Patients commonly present with symptoms from more than one phenotype [2]. The present case highlights a unique presentation in a pediatric patient with a longstanding aversion to solid food. Her eating challenges dated back to the attempted transition from breastfeeding to solids. Interviews with the patient suggested that eating difficulties were the direct result of fears of aversive consequences–specifically fear of choking–leading to functional impairment, emotional distress, and food avoidance.

While it is not uncommon for feeding and eating challenges to begin within a child’s first year of life [3] the prolonged duration of illness posed specific clinical challenges and risk factors, given the increased prevalence of psychological and functional impairments in older patients [4]. Further, due to her young age and lack of prior ability to articulate the underpinnings of her aversion to solid food, interventions for this case previously emphasized dietary-expansion focused food exposure rather than a fear-based exposure approach. Treatments that simultaneously address weight gain, growth, and medical complications of eating disorders alongside the resolution of food/eating-based phobias such as choking are scarcely available. The treatment manual for Cognitive Behavioral Therapy for ARFID (CBT-AR), published in 2019, outlines in vivo and interoceptive exposures to manage phobic responses to conditioned fears [5]. Through gradual, real-world exposure to less preferred foods and eating situations, as well as internal body sensations that trigger fear, children learn that they can tolerate discomfort and remain safe.

Strategic use of medication to optimize the benefits of therapy has shown promise in treating ARFID. Because ARFID is heterogeneous, medication choices should be guided by the specific phenotype (e.g., fear of choking vs. low interest vs. sensory sensitivity) and any comorbidities that medications might also target. There are no randomized trials of medication for ARFID; evidence is limited to case reports/series, often with mixed ARFID subtypes. In reports examining patients with ARFID and additional comorbidities (i.e. anxiety), small series suggest medications such as selective serotonin reuptake inhibitors (SSRIs), olanzapine, mirtazapine, and cyproheptadine may support weight gain and reduce distress [6–8].

For the fear of choking/fear of aversive consequences presentation, published cases most often describe SSRIs as first-line in both pediatric and adult choking phobia (with benzodiazepines sometimes added in adults) [9–12]. Some pediatric patients reportedly improved even after long-standing symptoms with low-dose SSRIs, possibly reflecting overlap with panic-spectrum mechanisms [10, 13]. Mirtazapine has also been reported to help restore solid intake after a choking incident in a child with acute refusal superimposed on chronic poor weight gain [14]. When SSRIs (and CBT) have not been sufficient to enable intake of solid food, case reports/series describe adding an atypical antipsychotic to reduce mealtime anxiety and rigidity—e.g., aripiprazole with rapid expansion of solid foods in one adolescent [15], and risperidone helping tube-fed children transition toward oral intake after SSRI failure or adverse effects [16].

## Case study

### Presentation

This 8-year-old female, “Angie,” presented for treatment never having transitioned to solid foods. From infancy, her mother noted that Angie would gag and often vomit when she introduced purees. A gastroenterologist was consulted at 15 months of age due to ongoing difficulties with intake, and an upper endoscopy identified eosinophilic esophagitis (EoE). A reduction in gagging and vomiting was noted after dairy was eliminated from Angie’s diet and ranitidine was initiated for several months, which was felt to be a sufficient intervention due to the relatively mild presentation of her EoE. Despite the dietary change, the patient remained unable to transition to solids; a dairy-free formula became the patient’s primary source of nutrition over the next two years. At age 4, Angie was presented with solid food again and parents noted a phobic response at that time. She was referred to occupational therapy (OT) and admitted to Children’s Hospital of Orange County inpatient feeding program for three weeks with limited progress noted. Angie began eating purees, but significant sensitivities to taste, texture, and smell persisted despite ongoing outpatient OT after the hospital admission. A second endoscopy at age 6 showed the EoE had resolved. She subsequently participated in outpatient therapy with a psychologist, during which a reduction in parental accommodation around food offerings was recommended. However, there was minimal improvement in her willingness to consume a broader range of foods. She was consuming only liquids and foods of a pureed consistency that did not require chewing at the time of admission to PHP.

Notable family history included maternal anxiety disorder and a sibling with picky eating tendencies. Angie and her family experienced chronic and significant interference with psychosocial functioning, including inability to eat sufficiently at school, in social settings, or outside of the direct care of her mother, who was spoon-feeding the patient to ensure adequate nutrition. Angie had significant linear growth suppression and a 2-year delay in bone age. She was at the 16th percentile for stature (44.5 inches) and at the 72nd percentile for BMI (weight was 48 lbs 10 oz). Despite her limited diet, she never required enteral feedings.

At admission, Angie reported no physical symptoms on a 14-point review of systems and specifically denied nausea, vomiting, early satiety, stomachache, gastroesophageal reflux, physical discomfort with swallowing, constipation, or diarrhea. Most labs were within normal limits, except for slight complete blood count abnormalities and mild elevations in lipids. Urinalysis was normal except for trace leukocyte esterase. The electrocardiogram showed normal sinus rhythm.

Angie did not have a history any psychiatric diagnoses beyond ARFID, and she was described as happy child who did not have notable anxiety outside of eating and dental appointments. Medically, she had strabismus which required surgery. She had no prior medication history except cyproheptadine for appetite stimulation.

### Course and outcome

Diagnostic challenges included the parents’ chief complaint and conceptualization of the maintaining mechanism as “picky eating.” Upon further assessment, it became apparent that while Angie was specific about presentation, taste, texture, and brands, those particular demands and desires were driven by the patient’s fear of choking rather than a true inability to tolerate a wide variety of tastes and textures as is seen in sensory-sensitive ARFID patients. Angie’s appetite was intact and she expressed marked interest in food, readily consuming without prompting those foods she deemed safe and low risk for choking.

Treatment included the following standard interventions within the PHP, five days per week: two meals and two snacks daily that were monitored and structured with therapeutic meal coaching (dinner and evening snack occurred at home), three times weekly medical monitoring of vital signs and weight, psychopharmacology and medication monitoring, individual therapy, group therapy consisting of psychoeducation and skills training and weekly family therapy. During a weekly multifamily treatment day, parents received six hours of interventions, including psychoeducation, experiential learning, process-oriented activities, a structured family meal, and multifamily group-based skills training. Treatment within the intensive outpatient program (IOP) was three hours daily, five days a week, and was gradually tapered to once weekly.

The primary treatment modality was family-based Cognitive Behavioral Therapy for ARFID (Table 1), supplemented by group therapies that incorporated skills from dialectical behavior therapy (for both parents and children), acceptance and commitment therapy (for children), and parent management training (for parents). CBT-AR is traditionally an outpatient intervention, and numerous adaptations were made to implement it within a higher level of care, including session frequency and length of stay. Stage 1 targeted necessary early behavioral changes prior to increasing food volume, such as the patient’s ability to arrive at the table without resistance or hiding and to feed herself independently. These interventions extended longer than typical at the PHP level of care. Stage 4 was extended due to the patient’s longstanding illness, the family’s difficulties consistently adhering to structure especially when Angie was distressed, and barriers identified within the family system (e.g., internalized weight bias, father’s limited involvement in treatment, and unhelpful comparison to sibling’s normative eating). This stage emphasized home-based strategies, generalization, parental efficacy, and maintenance of change, and included extensive training for Angie’s mother in the exposure protocols through in vivo coaching and support, as well as modeling through review of session video recordings.

**Table 1:** CBT-AR psychotherapy interventions modified for our partial hospital and intensive outpatient program

Month	Stage	Overview of Interventions	Objectives
1	Assessment	1. Pre-admission medical screening: vitals, physical exam, labs, ECG, and clinical interview to determine medical stability. 2. Post-admission clinical interviews with multidisciplinary treatment team (MD, therapist, RD, RN) and observations in milieu and therapeutic meals. 3. Family meal to evaluate symptoms and familial dynamics within the home. 4. Review collateral reports and medical records from birth to present.	Formulate initial conceptualization of disorder, family system, maintaining mechanisms and begin development of treatment plan. Identify any comorbidities impacting presentation and treatment outcomes.
	Family-Based CBT-AR: Stages 1 & 2	1. Family-based psychoeducation on ARFID diagnosis and anxiety. 2. Identify parent role in patient’s treatment and inadvertent maintenance of illness. 3. Initiate structured eating with mealtime rules and age-appropriate expectations. 4. Prescribe weight restoration meal plan and supplement protocol to prioritize nutritional compliance. 5. Identify and present primary maintaining mechanisms and the recommended treatment approach. Present rationale for exposure-based treatment: information on avoidance, safety behaviors, and maintaining mechanisms.	Demonstrate normative and age-appropriate mealtime behaviors, including arriving to table without resistance or avoidance and feeding self independently with supervision. Complete 100% of nutrition.
			Regulate eating and hunger with schedule and structure, including 3 meals and 3 snacks daily and achieve nutritional compliance with safe purees and nutritional supplement drinks to facilitate weight restoration process.
	Introduction to Parent Management Training	1. Present principles of behaviorism, highlighting concepts of behavior modification, including reinforcement and extinction. 2. Positive opposites identified to guide parental coaching. 3. Establish behavior reinforcement and contingency plan. 4. Support parents in consistent and effective implementation of behavior plan.	Develop insight into mechanisms of reinforcement that maintain avoidance, dysfunctional eating behaviors and psychosocial impairments.
2–6	Family-Based CBT-AR: Stages 2 & 3	1. Continue weight restoration, utilizing demand fading approach (increased portions of safe/preferred purees and nutritional supplements). 2. Maintain structure, rules and expectations. 3. Revisit psychoeducation and rationale for exposure as needed. 4. Initiate exposure therapy to target maintaining mechanisms.	Eliminate avoidance and develop understanding of the role of exposure in violating expectancies and learning new information.
	CBT: Psychoeducation, Identification of Thoughts, Examining Evidence & Checking Facts	1. Educate patient about normal body processes including mouth, tongue, throat and stomach, mechanisms of swallowing, in an age and developmentally-appropriate manner (e.g. Magic School Bus videos, child-friendly images of the GI tract). 2. Review diagrams of upper and lower gastrointestinal systems to recall how the esophagus functions, allowing food to pass through to induce cognitive dissonance during exposure sessions.	Develop understanding of the gastrointestinal system and its functioning, particularly the process of swallowing.
			Gain insight into fears, develop the ability to collect and evaluate data and fact-check.
	Inhibitory Learning	1. Generalize exposure learning from individual therapy sessions to dining room, home and school. 2. Gradual shift of demands from treatment team to family. 3. Vary day, time and location of exposures and increase the variety of food items presented. 4. Retrieval cues emphasized to maximize inhibitory learning.	Develop parental self-efficacy for management of eating disorder with fewer supports. Violate patient’s eating-based expectancies (e.g., eating = choking) and develop new associations which inhibit fear associations. Learn to cope with uncertainty and the presence of anxiety without avoidance.
	Parent Management Training	1. Modify targets and behavioral contingencies of plan. 2. Identify remaining patterns of positive and negative parental reinforcement. 3. Parents obtain in-vivo coaching at multifamily meals targeting avoidance patterns and parental responses to child’s emotional distress.	Create behavior change through intentional and timely positive reinforcement of desired behaviors. Parent gains insight and learns to cope with child distress as a result of creating structure, setting limits, and expectations.
7	Family-Based CBT-AR: Stages 3 & 4	1. Therapist engages family in weekly parent-led exposure sessions. 2. Parent observes weekly exposure sessions with individual therapist. 3. Parent learns Socratic questioning and follows scripts for processing exposures. 4. Exposure demand increases and interoceptive exposures are introduced alongside cue based exposures (food). 5. Identify patterns of parental anxiety and avoidance and develop a plan for management of family dynamics. 6. Present patterns of intermittent reinforcement to family and develop strategies for eliminating parental accommodation and inconsistencies. 7. Relapse prevention planning. 8. Coordination of care and referral to outpatient treatment.	Achieve greater learning, expectancy violation and inhibition of fear associations.
			Parental mastery of in-vivo skills coaching, exposure practices and management of avoidance behaviors.
			Prepare for risks of relapse and challenges outside of intensive treatment setting. Family linked with outpatient treatment providers based on clinical presentation at discharge.

Additionally, exposures were created using an inhibitory learning approach, given evidence that habituation alone was not effective in fear extinction. Due to actual bite exposures of food often occurring in the final few minutes of exposures to prevent escape/avoidance tendencies, the patient’s distress remained high throughout, thus habituation did not occur until the patient had actually swallowed and violated her expectancy of choking. This included identification of expectancies and violation of those beliefs, recall and retrieval cues, variation in therapists and day/time of exposure sessions, and combining feared cues with use of both food and interoceptive cues. See Table 2 for definitions of key behavioral principles, mechanisms, and intervention strategies in exposure-based CBT for ARFID.

**Table 2:** Glossary of key behavioral principles, underlying mechanisms, and intervention strategies in CBT-AR

Demand fading
A behavioral strategy in which expectations around eating are introduced gradually and increased over time. For example, a patient may initially be asked to interact with a feared food, then take a small bite, and later consume larger portions as tolerance improves.
Expectancy violation
A key mechanism of exposure in which the feared outcome does not occur, or occurs less severely than expected. In ARFID treatment, this may involve discovering that a feared food does not cause choking, vomiting, or intolerable sensory distress.
Generalization
The process of extending learning from exposures to new foods, settings, or contexts (e.g., eating a previously feared food at home, then at school or a restaurant).
Inhibitory learning approach
An exposure-based framework in which new, non-threat associations are learned that compete with (rather than erase) the original fear association. In ARFID, repeated exposure to feared foods helps patients learn that eating the food is safe and tolerable, weakening avoidance over time.
Interoceptive exposures
Exercises designed to intentionally evoke physical sensations that patients fear or avoid (e.g., fullness, gag sensations, throat sensations). In ARFID, these exposures help patients tolerate internal bodily sensations associated with eating.
Positive opposites
Adaptive behaviors that are incompatible with avoidance and restrictive eating and can be intentionally practiced and reinforced. Examples include taking developmentally appropriate bite sizes, trying new foods, and maintaining a typical eating pace.
Recall and retrieval cues
Strategies used to help patients remember and access learning from successful exposures across different settings. In ARFID treatment, cues (e.g., phrases, notes, photos of exposures, or specific coping statements) help patients recall that feared foods were previously tolerated.

Angie’s buy-in and motivational factors were important considerations throughout the course of care. When feeling overwhelmed by exposures around the midpoint of treatment, she expressed concerns suggesting a rupture in rapport and lessening motivation for the treatment approach. Angie’s statements included, “You guys don’t like me,” “You guys are wasting your time,” and “Why do I have to do exposures anyway?” Therapy providers repaired rapport by revisiting the rationale for the treatment approach, externalizing ARFID, aligning with the patient against ARFID, and practicing radical genuineness by openly expressing care for the patient, her functioning, and well-being. Behavioral contingencies, short- and long-term rewards, and subsequently consequences (such as completing extra therapy worksheets instead of attending a preferred group) were added to behavioral interventions to reduce escape/avoidance incidents and enhance motivation. Intermittent reinforcement due to non-adherence to the behavior plan was a concern and became a treatment target of Parent Management Training with Angie’s mother. Inconsistent adherence to the behavior plan was often driven by the aforementioned internalized weight bias, with her mother expressing concern around weight gain and the changes in Angie’s body shape/size. This was targeted via consistent psychoeducation on typical growth trajectories and reassurance that the temporary manifestations of weight gain (e.g., increased abdominal size) were a means to stimulate growth in height. Of note, Angie’s father’s lack of involvement in treatment often impacted behavior planning and contingencies by offering reinforcements without taking into consideration already agreed upon and implemented contingencies, which were utilized to target behaviors specific to the current phase of treatment. While providers worked to address this through video recordings of exposures and parent-only family therapy sessions to allow for revisiting the content and study skills/strategies utilized with the patient, it was evident that this alternative did not fully capture treatment, and a gap in knowledge, skills, and understanding remained. This noted gap between parents emphasizes the importance of having all caregivers involved in the treatment process.

Angie started PHP on cyproheptadine 2 mg nightly Sunday through Thursday, which she had taken in the past as an outpatient to help support appetite. The hope was that with a robust appetite, her willingness to taste new foods and textures would increase. Over the first month, fluoxetine was added and gradually increased for anxiety as it became clear that the most significant barrier to eating solids was a fear of choking and that low appetite was not as prominent a factor. Cyproheptadine was removed as fluoxetine was increased, as there is a known interaction between fluoxetine and cyproheptadine that may reduce fluoxetine’s effectiveness [17, 18]. While cyproheptadine is predominantly an antihistamine, it also antagonizes 5-HT2A and 5-HT1A receptors, which could interfere with the anxiolytic effects of SSRIs. During this time, she made significant progress in eating soft/mashable solids and began to consume new foods like pasta, cupcakes, and sliced avocado.

After 3 months of notable but slow progress, aripiprazole was added to augment the fluoxetine and help increase cognitive flexibility and promote change around the repetitive, intrusive fears of choking. Her mother felt that aripiprazole also increased Angie’s appetite, and Angie seemed more willing to try new, previously feared foods. During this time, she continued to make good progress and added new foods including sashimi, animal crackers, several fruits, and boiled eggs. Consistent with her fear of choking, she took larger bites of now safe foods, as well as smaller bites of feared foods. Angie was discharged to outpatient care on fluoxetine 40 mg and aripiprazole 2 mg, both of which appeared to be beneficial to her treatment course and well-tolerated. It was noted that the effective dose of aripiprazole may be higher than 2 mg as coadministration with fluoxetine (a potent inhibitor of CYP450 2D6) may significantly increase the plasma concentrations of aripiprazole [19].

During the course of treatment, which was 75 days over a 7-month period–she grew 1.5 inches in height and gained 13 pounds. She spent 6 weeks at PHP and the remainder of the time in IOP; it should be noted that the patient was stepped down to the IOP level of care earlier than desired by the treatment team and family due to insurance barriers.

Angie expressed pride in her progress over the course of treatment, particularly noting her improved ability to eat some of the same foods as her peers and family in social settings, as well as achieving her stated goal of eating a cupcake on her birthday.

## Discussion and conclusions

EoE is a recognized risk factor for ARFID and likely contributed to the early development of this patient’s feeding difficulties [20]. However, as in other pediatric cases with early medical and feeding complications, the relative contributions of physical discomfort, learned avoidance, and fear-based responding were difficult to disentangle retrospectively. In this case, although EoE may have played an etiologic role early in development, the patient’s later presentation appeared to be maintained primarily by fear of aversive consequences, specifically choking, rather than by ongoing medical symptoms.

This case also underscores the importance of careful diagnostic formulation in pediatric ARFID. Although the patient had long been conceptualized as a selective or “picky” eater and did show preferences related to texture, taste, and presentation, further assessment suggested that these features were more consistent with fear-based avoidance than with primary sensory sensitivity. As is common in ARFID, some overlap across phenotypes may have been present, but fear of choking appeared to be the predominant maintaining mechanism.

Over the course of treatment, the patient demonstrated substantial clinical improvement during a prolonged PHP/IOP admission. She expanded the range and texture of foods she could eat, improved in self-feeding and mealtime functioning, generalized gains across foods and settings, and showed meaningful physical growth. At discharge, while she was eating solids from all food groups, she continued to rely on purees and soft foods to fully meet nutritional needs. While patients with acute choking phobias often return to a fully unaccommodated diet following treatment, patients similar to Angie, who have a lifelong history of illness and no prior solid food intake, may not show a full resolution of symptoms during a single stay at a higher level of care. It is important to note that Angie continued to try new foods spontaneously until discharge, demonstrating confidence and willingness that far exceeded her baseline presentation at admission.

The treatment course suggests that CBT-AR adapted for a higher level of care may be feasible and clinically useful for young children with severe, longstanding ARFID characterized by fear of aversive consequences. Exposure work informed by inhibitory learning principles appeared to provide a useful framework in this case, particularly given that distress often remained elevated until expectancy violation occurred. However, caution is warranted in attributing improvement to any single treatment element. The patient received a multimodal intervention that included structured meal support, parent training, behavioral strategies, family therapy, repeated in vivo exposures, and psychopharmacologic treatment, precluding conclusions about the independent effect of inhibitory learning-based exposure specifically.

Similarly, this case suggests that PHP-level care can provide a valuable treatment context for severe pediatric ARFID, particularly when outpatient interventions have been insufficient and when families require intensive coaching to reduce accommodation and support exposures. However, conclusions regarding optimal level of care should remain tentative, as this single case does not establish superiority over less intensive settings. Fluoxetine and later aripiprazole appeared to coincide with gains in flexibility, willingness to approach feared foods, and overall progress, suggesting that adjunctive pharmacotherapy may have supported engagement in treatment. However, because medication changes occurred during ongoing intensive behavioral care, their specific effects cannot be determined.

In summary, this case describes meaningful improvement in a child with severe, longstanding ARFID who had never transitioned to solid foods. It suggests that CBT-AR adapted to a higher level of care, with intensive family involvement and exposure work informed by inhibitory learning principles, may be a useful approach for similar presentations. At the same time, the single-case design and multimodal nature of treatment limit causal inference. Further study is needed to clarify the role of higher levels of care, inhibitory learning-based exposure strategies, and adjunctive psychopharmacology in pediatric ARFID characterized by fear of choking.

## Data Availability

No datasets were generated or analysed during the current study.

## Abbreviations

ARFID: Avoidant/restrictive food intake disorder
CBT-AR: Cognitive behavioral therapy for ARFID
EoE: Eosinophilic esophagitis
IOP: Intensive outpatient program
OT: Occupational therapy
PHP: Partial hospitalization program
SSRIs: Selective serotonin reuptake inhibitors
